# Sleep’s role in updating aversive autobiographical memories

**DOI:** 10.1038/s41398-022-01878-1

**Published:** 2022-03-24

**Authors:** Yasmine Azza, Frank H. Wilhelm, Erich Seifritz, Klaus Junghanns, Birgit Kleim, Ines Wilhelm

**Affiliations:** 1 Translational Psychiatry Unit, Department of Psychiatry and Psychotherapy, University of Lübeck, Lübeck, Germany; 2 Department of Psychiatry, Psychotherapy and Psychosomatics, Psychiatric Hospital, University of Zurich, Zurich, Switzerland; 3 Division of Experimental Psychopathology and Psychotherapy, Department of Psychology, University of Zurich, Zurich, Switzerland; 4 Division of Clinical Psychology and Psychopathology, Department of Psychology, Paris-Lodron University of Salzburg, Salzburg, Austria

**Keywords:** Psychiatric disorders, Psychology

## Abstract

Aversive autobiographical memories play a key role in the development and maintenance of many mental disorders. Imagery rescripting is a well-established psychotherapeutic intervention aiming to create a more adaptive version of an aversive memory by modifying its interpretation. Sleep has been shown to support reconsolidation of updated neutral memories. Here, we investigated in healthy participants whether a 90-min nap compared to wake supports the adaptive reconsolidation of autobiographical memories. Forty-four university students received a single 50-min imagery rescripting session. Thereafter, half of the participants took a 90-min nap, whereas the other half stayed awake. Subjective (arousal ratings, reports of emotions and dysfunctional cognitions) and heart rate (HR) responses to individual memory scripts were measured before the intervention (pre), after the 90-min retention interval (post 1) and 7 days later (post 2). Results demonstrate a significant decrease in distress of aversive memories pre to post imagery rescripting. The nap group showed less distressing dysfunctional cognitions along with a lower HR in response to the negative memory script as compared to the wake group at post 1. These differences were no longer evident 1 week later (post 2). Central sleep spindle density during the nap was correlated with the reduction in HR in response to the negative memory script from pre to post 1. Our results provide first evidence for sleep benefitting adaptive reconsolidation of aversive autobiographical memories. Future research should expand this approach to clinical populations and investigate precise conditions under which sleep may benefit psychotherapeutic interventions utilizing reconsolidation mechanisms.

## Introduction

Most psychiatric disorders, such as posttraumatic stress disorder or anxiety disorders are characterized by aversive emotional memories [1]. This is prototypically known and well documented for posttraumatic stress disorder (PTSD), where the uncontrolled reexperiencing of traumatic episodes and the avoidance of trauma-related stimuli represent debilitating core features of the disorder [1]. In affective disorders, too, such as major depression, aversive memories represent a salient feature of the clinical picture, e.g., in form of negative memory biases and persistent intrusions of interpersonal crises or death [2, 3]. Current evidence-based treatments of such disorders employ exposure-based techniques to hamper the activation of such maladaptive memories via inhibitory learning [4, 5].

Imagery rescripting is a promising treatment that has introduced an innovative way of actively modifying maladaptive memories [6]. This treatment was designed to change the interpretation of aversive emotional memories by incorporating new adaptive information and feelings thereby reducing emotional distress associated with this memory [7]. The effectiveness of imagery rescripting has been demonstrated for different mental disorders such as PTSD and social anxiety disorder (for an overview, see [8]). During imagery rescripting, individuals are instructed to recall and re-experience their emotional memory by the use of mental imagery, which can activate similar neural structures as evoked by external stimuli and typically includes associated emotional responses [9]. Patients generate and imagine a less aversive course and outcome of the event (“script”), leading to modified, less distressing interpretations of the event and different emotions associated with it. Long-term therapeutic effects of imagery rescripting are assumed to be based on the subsequent reconsolidation of the updated memory [10].

While being relatively new in clinical psychology, the process of reconsolidation has been intensively studied in the field of basic neuroscience. Reconsolidation is defined as the re-stabilization of an updated memory [11–13]. More specifically, after its initial stabilization, referred to as memory consolidation, memories may re-enter a labile state when being reactivated during memory retrieval at a later point in time [11–13]. The capacity of a memory for temporary destabilization enables the incorporation of new information into the memory and in turn its modification [14]. Importantly, initial studies point toward a particular role of sleep, specifically slow-wave sleep and sleep spindles, in the re-stabilization of an “old” reactivated memory [15, 16]. In these studies, participants who had slept after the destabilization of an already consolidated memory showed better recall performance of the initial memory than those participants who stayed awake. A recent study goes beyond these earlier findings in demonstrating that sleep benefits not only the reconsolidation of the old memory trace but the integration of new information into the old reactivated memory [17]. In this experiment, participants first learned a set of paired objects which were later updated by reminding participants via contextual cues of the learned set right before a second set of objects was learned 2 days later. Total sleep time and spindle density in the subsequent night coincided with greater memory updating as indicated by an increased amount of second set objects that were remembered as first set objects. These findings are in concordance with earlier research describing a role for sleep spindles specifically in the integration of new declarative information into the network of long-term memories [18, 19]. These findings indicate a role of sleep in the reconsolidation of updated memories. However, it is unclear whether sleep also benefits the reconsolidation of aversive autobiographical memories, which appears to be of particular importance due to its clinical relevance.

Emotional memories comprise two components: the episodic content and the emotional tone that is associated with this content. A wealth of studies has provided evidence that sleep benefits the stabilization of the episodic content while results have been inconclusive with regard to the emotional tone. More specifically, some studies reported that sleep decreases [20, 21], or even increases [22] the emotional tone as measured by both subjective ratings and physiological response (i.e., heart rate (HR), HR deceleration, skin conductance response, positivity during late positive potentials). Thus, sleep’s impact on the emotional tone appears to be complex and discrepant results might in part be due to various factors such as the kind of measures of emotional tone and time after encoding (i.e., short—or long-term effects; see also [23] for a recent review on the role of sleep on emotional tone). For example, several recent studies reported time-dependent effects of sleep on emotional tone: while sleep after encoding preserved emotional tone in the short-term, it ameliorates emotional tone in the long-term [24–26]. It is important to note that the above-mentioned studies investigate the role of sleep on the initial consolidation of newly encoded emotional stimuli. Although an effect of sleep on the emotional response of an updated emotional memory appears to be very likely, this has not been investigated so far.

The current study investigated in healthy participants whether a 90-min nap following the modification of aversive autobiographical memories with imagery rescripting may benefit the reconsolidation of the rescripted memory. We decided to use a nap design because (1) napping has been shown to have a robust influence on memory processes that is comparable to a full night of sleep [27–30] and (2) in light of possible clinical applications, a nap appeared to be even more relevant as compared to a whole night of sleep as it is easier to be implemented in the clinical context. We hypothesized that the reduction in emotional distress in response to an individual aversive memory script as measured by continuous recordings of HR and subjective ratings of induced emotions and cognitions is greater when a nap as compared to a wake interval has followed memory modification. For the nap group, we further hypothesized a greater reduction in emotional response to the memory scripts in individuals exhibiting higher amounts of sleep spindles as well as a greater slow-wave activity (SWA, i.e., EEG activity at a frequency of 0.5–4 Hz which is characteristic of slow-wave sleep).

## Materials and methods

### Participants

Forty-six university students (bachelor, master, or Ph.D. studies) with a mean age of 23.16 (SD = 3.26) participated in the study. They received 75 Swiss francs for participation in the wake condition and 105 Swiss francs in the sleep condition. All participants provided written informed consent prior to participation. The study was approved by the local ethics committee (Ethics Committee of the Philosophical Faculty of the University of Zurich). The sample size was estimated by power calculations based on the effect of sleep vs. wake after therapy on symptom reduction of an earlier comparable study (two groups undergoing a therapeutic intervention with or without an ensuing 90 min sleep episode) [31]. One participant showed a clinically relevant level of depression as indicated by a Becks Depression Inventory [32] (BDI) score of 21 (predefined threshold for indicating clinically relevant depressive symptoms was set to ≥18) and was therefore excluded from the study. Another participant had to be excluded due to an insufficient amount of sleep during the nap interval (total sleep time of 13 min). The final sample used for statistical analyses comprised 44 Caucasian individuals (nap group: *n* = 21 (17 women) age: *M* = 22.14 ± 3.17 years; wake group: *n* = 23 (18 women) age: *M* = 24.09 ± 3.12 years).

### Procedure

**Fig. 1:**
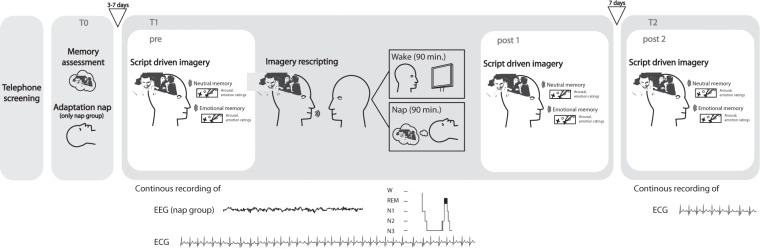
Study design. The study design included a telephone screening interview and three subsequent appointments at the sleep laboratory (T0, T1, and T2). At T0 the target memory for the imagery rescripting and a neutral memory were evaluated and all information necessary for the script preparation was acquired. Participants of the nap group also took an adaptation nap at T0. The second session (T1) included the script-driven imagery procedure (pre) followed by the imagery rescripting intervention while HR was continuously measured. Afterward, participants either took a nap (nap group) or watched an emotionally neutral movie (*Kingdom of the Blue Whale*, National Geographic; 2009) for a duration of 90 min (wake group). Another script-driven imagery assessment took place right after the nap/wake condition (post 1) and was repeated after seven days at T2 (post 2).

Prior to study start, interested participants underwent a telephone interview to screen for inclusion (age 18–30 years) and exclusion criteria (i.e., head injuries interfering with EEG measurements, neurological disorders, sleep disorders, drug intake, diagnosis of a mental disorder). In order to determine individual levels of depression and anxiety, the BDI [32] and the State-Trait Anxiety Inventory [33] was filled out by the participants online. The study design and procedures are summarized in Fig. 1. The first session (T0) consisted of a detailed assessment of an emotional as well as a neutral autobiographical memory. Only memories including an explicit perpetrator (i.e., someone perceived as causing emotional or physical harm to the participant) were used for the study. Subsequently, participants in the nap group underwent a 90-minute adaptation nap to familiarize themselves with the polysomnography recordings and the sleep laboratory environment, while the wake group was allowed to leave the sleep lab. After an interval of three to seven days, participants came back to the laboratory in the early afternoon for the experimental session (T1, including the pre and post 1 measurement).

After polysomnography, electrodes were attached for participants in the sleep condition (see below for details) and all participants completed the script-driven imagery procedure to evaluate both target memories (pre, see below for details). Thereafter, the imagery rescripting was conducted by a trained psychologist. Participants were then instructed to either take a 90-minute nap (nap group) or to watch an emotionally neutral film (*Kingdom of the Blue Whale*, National Geographic; 2009) during the same time interval (wake group). After this retention interval, participants underwent the script-driven imagery procedure again (post 1) to track any changes in the emotional response towards their individual memories. A third appointment (T2, post 2) took place seven days later to conduct a final script-driven imagery procedure identical to those conducted at the pre and post 1 measurement.

### Script-driven imagery

Script-driven imagery is a widely used and well-validated method to trigger emotional memories [34–36]. Participants were first asked to provide a detailed narrative of their neutral and aversive target memory. Then the experimenter prepared written scripts and audio recorded a 30-s narrative portraying both individual memories. The script-driven imagery task for each of the two memories consisted of four 30-s intervals (i.e., “baseline”, “script listening”, “script imagining”, “recovery phase”). During the “baseline” interval, participants were instructed to sit calm, close their eyes and try to relax. This “baseline” interval was followed by the exposure to the audio script (“script listening”). Thereafter, the participant was asked to imagine the event as vividly as possible (“Now, please imagine this memory as vividly as possible”) to introduce “script imaging” for both individual memories. During the subsequent “recovery phase” the participants were asked to disengage from the memory and relax. After each “recovery phase”, participants rated their subjective arousal experienced during the “imagery phase” of the script-driven imagery (1 = very calm to 10 = very aroused). The neutral memory script always preceded the aversive memory script to prevent spill-over effects. As a next step and only targeting the negative memory, the dominant emotion associated with the emotional memory was identified by the use of an adapted version of the Emotional Distress Inventory (EIBE [37]). The EIBE is an imagery rescripting-specific questionnaire, which asks participants to rate the activation of different negative basic emotions such as fear, shame or guilt (1 = not present to 10 = extremely strong) and the degree of distress associated with them (1 = not at all to 10 = very much). The most distressing emotion was defined as the “dominant emotion.” Further, the participant was instructed and supported by the experimenter to verbalize (dysfunctional) cognitions associated with the aversive memory and the degree of distress associated with the cognition (1 = not at all to 10 = extremely strong). The cognition entailing the greatest distress was later referred to as “dominant cognition”. Heart rate was continuously recorded during script-driven imagery.

### Imagery rescripting

The imagery rescripting procedure for the current study is based on the protocol of Schmucker and Köster [37] for PTSD patients and was conducted by a trained psychologist. Even though in the current study the intervention was conducted with healthy participants, the phenomenology of the target memories held similar features compared to those of traumatic memories. Namely, there was a perpetrator and the selected memories still elicited feelings of distress, helplessness, and injustice. For example, such memories consisted of bullying experiences, rejection in a relationship or being falsely accused. Before the start of the intervention, participants were informed about the course and aim of the imagery rescripting procedure. After this introduction, participants were instructed to close their eyes or (if this was uncomfortable) to look to the ground during the intervention. A 5-min breathing exercise was conducted for relaxation before the actual intervention.

In *phase 1* (“imaginal exposure”), participants were asked to imagine the selected memory as vividly as possible while reporting it to the experimenter in as much detail as possible. If necessary, this process was supported by the experimenter asking questions such as: “What do you see?”, “What do you smell?”, “What do you hear?”. Afterward, the participant was asked to go back to the beginning of the scene and recount the aversive experience up to the most distressing part of the memory, the so-called “hotspot” of the event. Introducing *phase 2* (“mastery imagery”), the participant was asked to imagine the current self as a bystander observing the scene happening to the younger self. He/she was asked to describe feelings and perceptions from this new perspective. The participant was asked if any questions, thoughts or wishes for actions towards the perpetrator have arisen. He/she was further encouraged to pose these questions or thoughts or execute these actions directed toward the perpetrator in their imagination. A conversation between the current self and the perpetrator proceeded until any sign of disempowerment of the perpetrator was described by the participant, e.g., the perpetrator feeling shame. In *Phase 3* (“self-calming”), the current self directly interacted with the younger self thereby offering consolation and explanations, typically associated with a sense of relief. Finally, the participant was encouraged to imagine for 30-s a positive closing image (i.e., the personally most meaningful and important image of the whole session) that aimed at terminating the intervention with a sense of completion. The duration of the intervention varied depending on the length and complexity of the individual memory as well as the individual time needed for successful memory modification (which was mostly limited by the time participants needed for disempowerment of the perpetrator) with a mean duration of 50 min and a range of 25–80 min. During the intervention, HR was continuously recorded, and ratings of arousal were repeatedly assessed at relevant points, i.e., for start, hotspot, disempowerment of the perpetrator, interaction with the younger self and closing image. Before each rating a marker was set in the HR recording for the later recapitulation of the intervention phases.

### Polysomnography

Sleep was recorded using a portable EEG device (SOMNOscreenTM plus, SOMNOmedics GmbH, Randersacker, Germany). Fourteen Ag/AgCl electrodes were applied to the cleansed sites at the scalp, face and upper body of the participants, including Fz, C3, C4, Cz, Pz, Oz, A1, A2, Ground, Electrooculography (left and right), Electromyography (left and right upper chin and beneath chin), Electrocardiography (ECG) (Lead II: left lower rib cage and right clavicle), according to the AASM recommendations [38]. EEG recordings were referenced to Cz. All channels were sampled at 256 Hz. To ensure high quality, impedances were kept within a tolerable range (≤20 Ω). Sleep measures of the nap group resemble typical values of nap studies and are summarized in Table 1.

**Table 1:** Descriptive sleep measures of the nap group.

	Duration in minutes Mean (SEM)	Percentage of total sleep time Mean (SEM)
Total sleep time	71.95 (3.74)	
NREM1 sleep	7.38 (1.18)	10.15 (1.41)
NREM2 sleep	35.76 (2.76)	51.25 (3.81)
NREM3 sleep	16.14 (3.17)	22.91 (4.49)
REM sleep	6.57 (1.79)	7.92 (2.17)
WASO	6.0 (1.69)	7.64 (2.10)

*REM* rapid eye movement sleep, *NREM* non REM sleep, *WASO* wake after sleep onset.

### Data reduction and statistical analysis

ECG data were analyzed using the software Autonomic Nervous System Laboratory (ANSLAB [39, 40] version 2.6). In brief, ECG data were resampled to 400 Hz using cubic spline interpolation and low-pass filtered at 40 Hz. R-waves were then automatically detected, visually inspected and manually edited for occasional misdetections, ectopic beats and artifacts. Heart period was calculated as the interval in milliseconds between successive R-waves. For ease of interpretation, heart period was converted to instantaneous HR (in beats per minute, bpm) using the formula HR = 60,000/heart period and resampled to 4 Hz resolution for precise segmenting.

For imagery rescripting, the following segments were extracted for 20-s intervals preceding the respective markers (except for the initial interval which succeeded the start marker) set by the psychologist during the imagery rescripting session: 1) start of the intervention, 2) hotspot, 3) disempowerment of the perpetrator, 4) positive interaction, and 5) closing image. Mean HR was calculated for each of these segments.

In order to calculate HR response to the aversive memory script for each of the three sessions (pre, post 1, post 2), HR change scores were calculated by subtracting the 30-s “script imaging” intervals of the neutral from the emotional memory scripts. HR data count was reduced due to technical artefacts (*N* = 1 at post 1) or missing values (*N* = 1 at pre, *N* = 1 at post 1, *N* = 2 at post 2). HR data of four participants exceeding 1.5× the interquartile range of the HR change score (emotional minus neutral) sample distribution were further excluded from the analyses [41]. However, including these outliers did not essentially change the pattern of results.

EEG signals were re-referenced to mean activity of both mastoids and filtered between 0.016 and 35 Hz. Electroencephalographical sleep recordings were manually scored according to American Academy of Sleep Medicine (AASM) criteria [38] using the software SchlafAus 1.0 (developed by Steffen Gais, unpublished, University of Tuebingen, Germany). Fast sleep spindles (13–15 Hz) were automatically detected during stage N2 and N3 using the SpindleToolbox [19] in Matlab 2016b (The MathWorks, Natick, MA). For each participant the individual sigma power spectrum was plotted, which allowed for visual identification of the individual peak of the frequency band. The central electrode Cz was used for analysis due to the dominance of spindle events over the central cortex [42] as well as the fact that central spindle activity has been associated with memory processing [43] and affective processing [44].

A 3 × 2 × 2 analysis of variance (ANOVA) was computed in SPSS v.23 (IBM Corp., Armonk, NY, USA) with “Time” (pre, post 1, post 2) and “Valence” (emotional, neutral) as within- and “Group” (sleep, wake) as between-subject factors for each main outcome (HR, dominant emotion and cognition). Levene’s test for homoscedasticity was conducted to assure variances between groups were comparable. In case sphericity assumptions were violated, Greenhouse–Geisser corrected values are reported. Pearson’s correlation coefficient (or Spearman’s rank correlation coefficients in case of violation of the normality criterium) as well as dependent *t*-tests (or Wilcoxon-tests if the data did not fulfill the requirements for a *t*-test) were calculated using R (version 3.3.3 [45]).

## Results

### Emotional reactivity during imagery rescripting

**Fig. 2:**
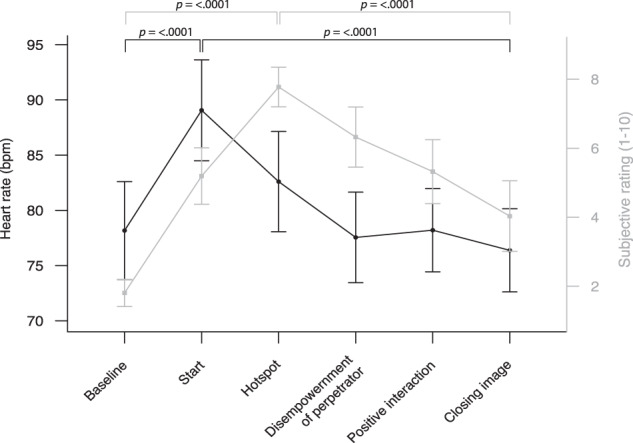
Heart rate and self-reported arousal values during imagery rescripting. Mean HR (bpm), respective standard errors (lines and scale in black) and self-reported arousal ratings (lines and scale in grey) at different time points during the imagery rescripting session (i.e., baseline, start, hotspot, disempowerment of perpetrator, positive interaction and closing image).

Emotional reactivity was tracked throughout the imagery rescripting procedure using arousal ratings as well as HR as a more objective indicator of psychophysiological arousal. The trajectories of these two measures during imagery rescripting differed throughout the imagery rescripting phases (see Fig. 2). While subjective arousal increased from baseline to the hotspot of the aversive memory (*z* = −1.28, *p* < 0.0001) and decreased towards the end of the intervention (*t*(44) = 9.52, *p* < 0.0001), HR showed an increase after the baseline with peak level at the start of the intervention (*t*(43) = −8.38, *p* < 0.0001) and a steep decrease to the closing image (*t*(41) = 8.77, *p* < 0.0001). Due to the divergence of self-reported arousal and psychophysiological HR response in the current study, we focused on HR as primary arousal measure in subsequent analyses. Heart rate is a well-established measure of psychophysiological arousal with large effect sizes for discriminating, e.g., fearful from non-fearful situations and participants [46]. Further reasons for focusing on this variable were: 1) subjective arousal to be more biased due to expectancy and social desirability effects, 2) better linear scalability of HR and 3) segmented HR intervals represent a continuous measure during a specified time period, while arousal ratings were elicited occasionally by interrupting the imagery rescripting process and were in part retrospective.

### Effects of sleep on the emotional response to the aversive memory

#### Heart rate response

**Fig. 3:**
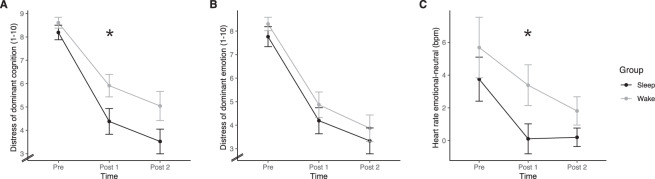
Emotional response to the aversive memories. Distress of dominant cognition (**A**), distress of dominant emotion (**B**), HR response in bpm (emotional–neutral) (**C**) for the sleep (black lines) and wake group (grey lines) across the three timepoints (pre, post 1, post 2). Heart rate response was computed by subtracting mean HR during imagery of the neutral memory script from mean HR during imagery of the aversive memory script; the degree of induced distress ranged from 1 = not at all to 10 = extremely strong. *p < 0.05.

The emotional response to an individual aversive and a neutral memory script was measured before the intervention (pre), immediately after the retention interval (post 1) and 1 week later (post 2) using script-driven imagery. Heart rates were significantly higher when imagining the aversive memory script as compared to the neutral memory script (main effect Valence: *F*(1,34) = 16.20, *p* < 0.001, $n_{\it{p}}^2 = 0.26$). However, this effect was moderated by the time of assessment (interaction Valence × Time: *F*(1.86,63.15) = 4.71, *p* = 0.014, $n_{\it{p}}^2 = 0.12$). In order to further explore this interaction effect, we performed 2 × 2 ANOVAs for each of the three time points separately. Before the intervention (pre), HR was significantly higher during imagery of the aversive compared to the neutral memory script (main effect Valence (*F*(1,37) = 26.56, *p* < 0.001, $n_{\it{p}}^2 = 0.42$) and both, the sleep and wake group did not differ from each other (main effect Group: *p* > 0.90; interaction Valence × Group: *p* > 0.78). After the retention interval (post 1), we found a significant interaction effect between the factors Valence and Group (*F*(1,36) = 4.75, *p* = 0.036, $n_{\it{p}}^2 = 0.12$; Fig. 3C). More specifically, for participants who were allowed to take a short nap after the intervention, HR was comparable during imagination of the aversive and the neutral memory script at post 1 (aversive: *M* = 73.97, SEM = 2.19; neutral: *M* = 74.10, SEM = 2.30; *p* = 0.88). In those participants who stayed awake, HR at post 1 was higher for the aversive as compared to the neutral memory script (aversive: *M* = 71.47, SEM = 2.48; neutral: *M* = 68.90, SEM = 2.22; *p* = 0.011). One week later, HR did not differ between the aversive and the neutral memory script and the interaction between the factors Valence and Group was no longer significant (main effect Valence: *p* = 0.129; interaction Valence × Group: *p* = 0.223).

#### Emotions and cognitions

Participants varied regarding the primary distressing emotion being activated (sadness, *n* = 9; powerlessness, *n* = 8; guilt, *n* = 8; anger, *n* = 7; shame; *n* = 6; loneliness, *n* = 4; resignation, *n* = 1; horror, *n* = 1). For further analysis, we determined for each individual the intensity of the most distressing emotion and the subjective level of distress of the most dominant dysfunctional cognition referred to as “dominant emotion” and “dominant cognition”. As Fig. 3A indicates, subjective distress related to the dominant dysfunctional cognition showed a strong decrease after the intervention (main effect of Time: *F*(1.64,68.95) = 74.3, *p* = <0.001, $n_{\it{p}}^2 = 0.64$). No significant interaction effect was found for the factors Group and Time (*F*(1.64,68.95) = 1.61, *p* = 0.2). Sleep and wake groups differed significantly with regard to the distress of dominant cognition (main effect Group: *F*(1,42) = 4.69, *p* = 0.036, $n_{\it{p}}^2 = 0.10$). Exploratory *t*-tests indicated that groups did not differ significantly before the intervention (Wake: *M* = 8.61, SEM = 0.23; nap: *M* = 8.19, SEM = 0.31, both *p* > 0.28). However, distress related to the dominant cognition was significantly lower at post 1 in the sleep as compared to the wake group (wake: *M* = 5.91, SEM = 0.48; nap: *M* = 4.38, SEM = 0.55, *p* = 0.042). At post 2, distress of the dominant cognition was only marginally different between the groups (wake: *M* = 5.04, SEM = 0.62; nap: *M* = 3.52, SEM = 0.53, *p* = 0.073, see also Fig. 3A). Concordantly, the intensity of the dominant emotion strongly decreased after imagery rescripting as indicated by a significant main effect of Time (*F*(1.55,65.05) = 112.31, *p* = <0.0001, $n_{\it{p}}^2 = 0.73$; Fig. 3B). No significant interaction effect was found for the factors Group and Time(*F*(1.55,65.05) = 0.34, *p* = 0.657) and sleep and wake groups did not differ regarding the distress related to the dominant emotion (*F*(1,42) = 0.94, *p* = 0.337). Exploratory post-hoc *t*-tests for the dominant emotion indicated that groups did not differ significantly before the intervention (dominant emotion: Wake: *M* = 8.3, SEM = 0.28; sleep: M = 7.76, SEM = 0.42) or at any later point in time.

### Association between sleep physiology and emotional response

**Fig. 4:**
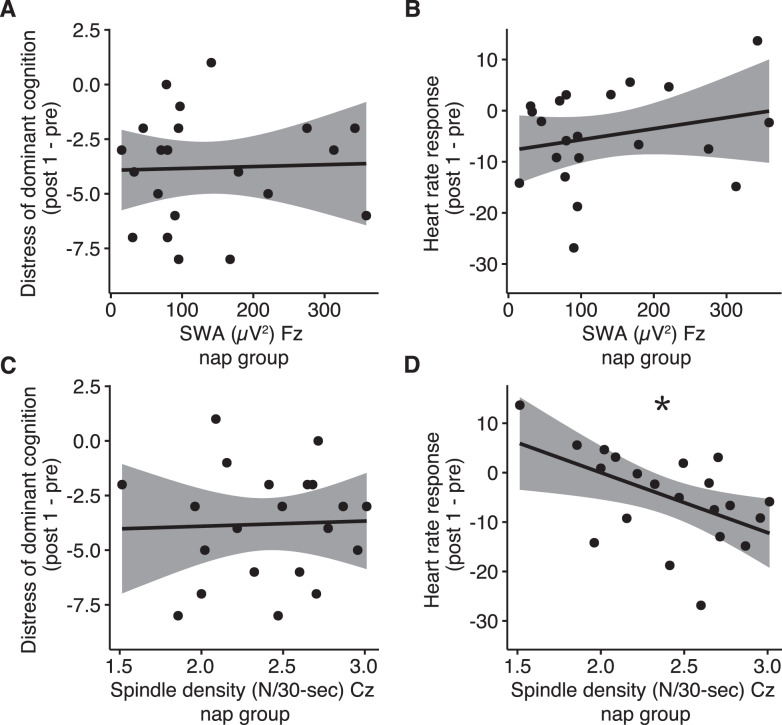
Correlation between sleep indices and reduction of arousal and distress. Scatter plots illustrating the correlation between sleep physiology (SWA and fast spindle density) and reduction of distress associated with the aversive memory (**A**, **C**) and arousal measured by HR (**B**, **D**) from pre to post. The 1.95% confidence intervals are depicted in grey. *p < 0.05. SWA = slow wave activity (measured at Fz in the spectral band 0.5–4 Hz); fast sleep spindles (13–15 Hz) were measured at Cz and automatically detected during stage N2 and N3.

To further explore the role of sleep in the reconsolidation of aversive memories, we correlated physiological sleep indices with those measures of emotional response showing group differences (i.e., distress of dominant cognition and HR response). Based on previous research indicating a role of SWA and sleep spindles in memory reconsolidation [15, 17], we included these two measures in the correlation analyses. Neither the reduction in distress of the dominant dysfunctional cognition nor HR response (i.e., the difference in HR when imaging the aversive versus the neutral memory script) significantly correlated with frontal SWA (cognition: *r* = 0.06, *p* = 0.81; HR: *r* = 0.15, *p* = 0.52; Fig. 4A, B). Sleep spindle density was not correlated with distress of the dominant cognition (*r* = 0.04, *p* = 0.87; Fig. 4C). Notably, spindle density during the nap predicted reduced HR response at post 1 (*r* = −0.55, *p* = 0.013, Fig. 4D).

## Discussion

The current study examined whether a 90-min nap compared to wake supports the reconsolidation of an updated autobiographical memory. In line with previous research in psychiatric patients (e.g. [47–50]), the present work demonstrates that imagery rescripting decreased dysfunctional cognitive and emotional responses to an idiosyncratic aversive memory formed earlier in life. Importantly, our results indicate that a short interval of sleep after imagery rescripting enhances its beneficial effect as indicated by a lower HR response and less distressing dysfunctional cognitions associated with the aversive autobiographical memory script. This immediate advantage of the nap over the wake group was no longer evident 7 days later. Fast central sleep spindle density (13–15 Hz) during the nap predicted the sleep-related reduction in HR.

During imagery rescripting, an individual aversive memory is made labile and is updated with novel adaptive information, which in the subsequent process of reconsolidation is assumed to become stabilized and reintegrated into long-term autobiographical memory networks [7]. In those participants who were allowed to take a short nap but not those who stayed awake after imagery rescripting, HR response to individual memory scripts were no longer increased for the aversive compared to the neutral memory script. Thus, a period of sleep, as compared to wakefulness, led to a greater reduction of the emotional tone associated with an aversive memory suggesting that this memory has become “neutral.” In parallel to the sleep-related effect on psychophysiological response, the subjective distress associated with the dysfunctional cognition activated by the negative memory script was also found to be lower after sleep as compared to wakefulness. This effect reached significance only in an exploratory t-test but not in the overall ANOVA and should therefore be interpreted with caution. In sum, these findings are in line with previous studies showing that sleep supports memory reconsolidation in humans [15–17] as well as in animals [51]. Importantly, they go beyond these earlier findings in showing that sleep benefits an adaptive reconsolidation-updating of longstanding and well-consolidated negative autobiographical memories.

The active system consolidation hypothesis postulates that the repeated reactivation of newly acquired memories during sleep essentially contributes to the integration and stabilization of newly acquired hippocampal memory representations. Slow wave activity, i.e., oscillatory EEG activity characteristic for deep sleep, is assumed to drive this reactivation process in the hippocampus in temporal coordination with thalamo-cortical sleep spindles inducing synaptic plasticity in the neocortex [52, 53]. Thus, slow waves and sleep spindles are critically involved in the process of memory consolidation. The neurophysiological mechanisms underlying the beneficial effect of sleep on memory reconsolidation are proposed to overlap with those mechanisms underlying the consolidation of memories during sleep [15, 17]. Our finding of sleep spindle activity predicting the reduction of psychophysiological arousal induced by an aversive memory script is not only in line with this theoretical assumption, it fits well with previous studies showing that sleep spindles are particularly involved in the integration of new information in the long-term network of neocortical memories [18, 54, 55]. Moreover, this finding is also in line with earlier research showing that sleep spindle density is associated with successful updating of neutral memories [17]. Of course, as our finding is of correlative nature, assigning a causal role to sleep spindles in autobiographical memory updating is plausible but somewhat speculative. Future studies are needed that aim at disentangling the underlying causal mechanisms by modulating spindle activity, e.g., by the use of pharmacological means [56] or by auditory or electrical stimulation [57, 58].

Beneficial effects of sleep on the emotional response towards a negative autobiographical memory were found immediately after the nap, but sleep and wake groups no longer differed 1 week later. This pattern of results stands in contrast to recent studies showing that sleep after encoding of novel emotional stimuli helps to immediately preserve the emotional tone associated with the episodic content while it ameliorates the emotional tone in the long-term [58–61]. In these studies, it has been hypothesized that sleep initially acts to consolidate the emotional salience of memories which helps further processing of the memory and ultimately leads to a reduction of the affective tone in the long-term. Importantly, these findings merely refer to the process of consolidation whereby a memory trace must be newly established. In the current study, a well-consolidated trace of a past memory becomes activated and updated with novel information, and this may accelerate the above-mentioned processes of emotional memory processing. This assumption is well in line with previous findings demonstrating that existing memory networks facilitating the incorporation of newly associated information can accelerate sleep-dependent processes of consolidation [60]. However, a possible acceleration of emotional processing due to the updating of old memories does not sufficiently explain why the effects of sleep on the reduction of the emotional tone were no longer evident one week later. The missing long-term difference may not be due to the sleep group having lost its advantage but the wake group having managed to catch up. We hypothesize that the temporally-delayed reduction of emotional response in the wake group is due to further sleep intervals occurring during the 7-day retention interval. Previous research has not conclusively specified the exact time window during which sleep must occur after learning to enhance memory consolidation and reconsolidation. With regard to memory consolidation, the length of this critical time window seems to depend on the type of memory task [61] with critical intervals of 3 h reported for declarative memory consolidation [62, 63] and up to 12 h for procedural memories [64, 65]. Basic neuroscience findings point toward a critical time window also referred to as “reconsolidation window” of 5 min up to 6 h after reactivation during which the memory remains labile and vulnerable to interventions targeting the process of re-stabilization (e.g., [66]). In total, our findings of immediate but not long-term effects of a nap suggest that sleep can support memory reconsolidation to some extent even when it occurs several hours after memory modification. Future research is required to systematically test the critical time window of sleep occurrence after reactivating autobiographical memory content longitudinally over several days.

We report evidence for an effect of a short daytime nap on HR and distress of the dominant dysfunctional cognition, whereas no effect was found for the intensity of the dominant emotion. One may speculate that HR might per se be more sensitive to uncover small effects of sleep on the characteristics of a memory trace as we did not find a significant agreement between arousal ratings and HR values. Moreover, the specific assessment of emotions and cognitions which substantially differ between each other and between participants might have played a role in this context. While the dominant cognition was identified in the course of the detailed conversation and elaboration with the psychologist, the dominant emotion and its distress was evaluated by a questionnaire asking the participant to mark the most prevalent emotion from a list of basic emotions, such as sadness, anxiety and anger. Participants may have had difficulties to evaluate and recognize their dominant emotion in a mix of experienced emotions. Assessing one’s affective states represents an abstract and biased task as also discussed in Haybron [67], who analyzed and questioned the reliability of individuals’ evaluation of their affective states. It appears that an informed and reasonable selection of outcome measures best characterizing the individual autobiographical memory trace is fundamental and requires further research. The utility of other objective measures besides HR, including psychophysiological, neuroendocrine and neural markers should be evaluated in future research.

A limitation of the present study lies in the entirely Caucasian and predominantly female sample. Future studies should include more diverse populations to determine the utility of the intervention. Further, imagery rescripting was selected in this experiment as a therapeutic technique that is assumed to initiate reconsolidation processes [7, 68]. We did, however, not directly test and confirm that the beneficial effect of sleep on imagery rescripting depends on reconsolidation mechanisms. Alternative theoretical accounts of possible mechanisms underlying the effect of imagery rescripting (i.e., “retrieval competition account” and “extinction”) postulate that imagery rescripting works by creating a new adaptive memory trace that competes with the original [69] rather than modifying an old memory trace. These alternative explanations are also consistent with the current state of basic research as sleep has been found to support not only reconsolidation but also the consolidation and extinction of aversive memories [21]. Future studies could include a “no-reactivation” or a “no-modification” condition which would be necessary control conditions for drawing firm mechanistic conclusions about reconsolidation processes.

In sum, here we could demonstrate that a short nap after reactivating and modifying a personal relevant, aversive memory using imagery rescripting reduces the emotional distress related to this memory indicating that sleep supports the efficiency of imagery rescripting. These findings are of high clinical relevance as they suggest that sleep may benefit the efficacy of psychotherapeutic interventions which implicitly or explicitly take advantage of (re)consolidation mechanisms targeting emotional autobiographical memories. About half of patients suffering from mental disorders receiving psychotherapy do not substantially reduce their symptoms [70]. Disturbed sleep which is highly prevalent in mental disorders might be one factor limiting psychotherapeutic success [71]. Previous research indeed indicates that poor sleep quality is related to lower benefits of psychotherapy [72]. Our findings encourage future research considering and testing sleep augmentation as a novel treatment component to enhance established psychotherapeutic interventions. This idea has recently been discussed by us and others [73–75] but empirical evidence is so far scarce.
